# An Acid Up-Regulated Surface Protein of *Lactobacillus paracasei* Strain GCRL 46 is Phylogenetically Related to the Secreted Glucan- (GpbB) and Immunoglobulin-Binding (SibA) Protein of Pathogenic Streptococci

**DOI:** 10.3390/ijms20071610

**Published:** 2019-03-31

**Authors:** Susan J. Pepper, Margaret L. Britz

**Affiliations:** 1 Monash Sustainable Development Institute, 8 Scenic Boulevard, Monash University, Clayton, VIC 3800, Australia; sue.pepper@monash.edu; 2 Centre for Food Safety and Innovation, Tasmanian Institute of Agriculture, Life Sciences Building, University of Tasmania, Hobart, TAS 7005, Australia

**Keywords:** *Lactobacillus casei*, *Streptococcus*, SibA, CHAP, PcsB, cell-wall hydrolase, acid resistance

## Abstract

Bacterial cell wall hydrolases, including amidases and peptidases, play a critical role in peptidoglycan turnover during growth, impacting daughter cell separation, and cell death, through autolysis. When exploring the regulation of protein expression across the growth cycle of an acid-resistant strain of *Lactobacillus paracasei*, GCRL 46, we observed temporal up-regulation of proteins in the 40–45 kDa molecular weight range for whole-cell extracts when culturing in fermenters at a controlled pH of 4.0 versus optimum growth pH of 6.3. Up-regulation of proteins in this size range was not detected in SDS-PAGE gels of the cytosolic fraction, but was routinely detected following growth at low pH in whole cells and cell debris obtained after bead beating and centrifugation, indicating a cell surface location. N-terminal sequencing and in silico analyses showed sequence similarity with proteins in the *L. casei* group (*L. casei*, *L. paracasei* and *L. rhamnosus*) which were variously annotated as uncharacterized proteins, surface antigens, possible TrsG proteins, CHAP (cysteine, histidine-dependent amidohydrolases/peptidases)-domain proteins or putative peptidoglycan d,l-endopeptidase due to the presence of a CwlO domain. This protein is a homologue of the p40 (Msp2) secreted protein of *L. rhamnosus* LGG, which is linked to probiotic functionality in this species, and is phylogenetically related to structurally-similar proteins found in *Enterococcus*, *Streptococcus* and *Bifidobacterium* species, including the glucan-binding (GbpB), surface antigen (SagA) proteins detected in pathogenic group A streptococci species as secreted, immunoglobulin-binding (SibA) proteins (also named PcsB). Three-dimensional (3D) modelling predicted structural similarities in the CHAP proteins from the *L. casei* group and streptococcal species, indicating retention of overall architecture despite sequence divergence, and an implied retention of function during evolution. A phylogenetically-related hydrolase also contained the CwlO domain with a NLPC_P60 domain, and showed similar overall but distinct architecture to the CHAP proteins. We concluded that the surface-located, CHAP protein in *L. casei* is up-regulated during long-term exposure to acidic conditions during growth but not during acid shock.

## 1. Introduction

The gram-positive lactic acid bacteria (LAB) belong to several genera in the phylum *Firmicutes*, including *Lactococcus*, *Enterococcus*, *Oenococcus*, *Pediococcus*, *Streptococcus* and *Lactobacillus*. LAB have in common the ability to convert sugars into lactic acid either homo- or hetero-fermentatively (the latter potentially producing ethanol, aldehydes and mixed acids in addition to lactic acid). They occupy a variety of ecological niches in nature, notably associated with human and animal gastrointestinal tracts (GIT) and mucosal surfaces, and have been used traditionally in the production of fermented meat, plant and dairy products [1,2]. Fermented foods are preserved through acidification, and LAB help develop flavour and aroma qualities in food, particularly during the ripening phases of fermented milk products [3,4]. *Lactobacillus* comprise one of the largest genera of the LAB, with 237 species currently reported in the list of prokaryotic names with standing in nomenclature (http://www.bacterio.net/lactobacillus.html). Several *Lactobacillus* species are used in food fermentations as starter cultures, which have a primary function of rapid acidification of the food matrix, while others occur as non-starter LAB (NSLAB) which develop as part of the food microbiota in maturing cheeses [5]. While comprising a minor part of human GIT microbiota [6,7], probiotic species of *Lactobacillus* confer human health benefits [8,9,10], with documented impacts involving immunostimulation and antagonism towards pathogenic microbes through competitive exclusion, which involves their ability to attach to GIT epithelium cells and mucins [11,12]. Many of the probiotic traits of *Lactobacillus* species are associated with cell surface structures and surface plus secreted proteins [13,14,15]. The cell wall forms a physical barrier outside the cytoplasmic membrane and is involved in functions associated with responding to external environmental stimuli [13] and determining cell shape plus integrity [14]: it is essential for cell survival.

The cell wall of *Lactobacillus* consists of the peptidoglycan (PG) sacculus, which is made up of glycan chains containing *N*-acetylglucosamine and *N*-acetylmuramic acid that are attached to short peptides which cross-link to form a mesh-like structure. In addition to potential modification to the PG by *O*-acetylation or *N*-deacetylation, the surface can be decorated by teichoic acids and other surface polysaccharides [13]. Some species of *Lactobacillus* also synthesize an S-layer, which is an outer microcrystalline protein sheath which has been implicated in probiotic function, including infection control and adhesion [16,17,18]. The formation of the cell surface is a dynamic process, requiring multiple enzyme systems in the synthesis of chemical components and their assembly into the sacculus during growth, which involves turnover of the structures during elongation, septum formation and daughter cell separation [13]. PG (murein) hydrolases are a diverse group of enzymes (amidases, peptidases, muraminidases and glucosaminidases) [13] responsible for cleaving specific bonds in PG during turnover and can also lead to cell lysis [19]. Starter culture autolysis during food fermentations can aid the release of cytoplasmic enzymes involved in flavour development and provide nutrients for metabolism by NSLAB during cheese ripening or product storage, which also contributes to organoleptic qualities [20,21].

Secreted proteins detected in the extracellular culture medium of the probiotic *Lactobacillus rhamnosus* strain LGG were shown to prevent cytokine-induced apoptosis and regulate cell growth and survival in intestinal epithelial cells, as well as ameliorate intestinal inflammation in mice [22,23,24]. These proteins were originally named p75 and p40 (based on apparent molecular weight, MW) but later renamed major secreted proteins (Msp1 and Msp2 respectively), with homologues detected in *L. casei* BL23 [25,26,27]. Bäuerl et al. [27] demonstrated muropeptide hydrolysis by p75/Msp1 in *L. casei* BL23 and used knock-out mutants to conclude that this protein was linked to cell separation, given that mutants formed elongated cells. Similar observations were later made for strain BL23 using immunolocalization to demonstrate Msp1 at cell poles during septation and to further demonstrate that PG was cleaved in the stem peptide, indicating d,l-endopeptidase activity [26]. Knock-out mutants for Msp2/p40 in strain BL23 showed no impact on growth [26], whereas similar knock-out mutants were not obtained in *L. rhamnosus* likely due to the essential nature of this protein in this species [25]. Several other putative PG hydrolases were identified from in silico analysis of the sequenced genome of BL23, including the Msp1 protein (which had a predicted MW of 49.6 kDa), where some were predicted as encoded by prophages [26]; the anomaly in the difference in MW of Msp1 experimentally detected in *L. rhamnosus* LGG and the cloned protein expressed in *Escherichia coli* was later attributed to glycosylation of the Msp1 protein in these species but not in *L. casei* [28].

While characterizing the acid tolerance of a dairy strain of *L. paracasei*, GCRL 46, we noted the up-regulation of cell-associated proteins following culture under controlled fermentation conditions at low pH. We argue that these culture conditions may better represent how cells are exposed to acidic environments during food processing and product storage in the dairy industry (noting that yogurt pH is normally 4–4.6 and cheese pH is 5.1–5.5, https://www.foodqualityandsafety.com), in contrast to acid shock conditions where there is a considerable and established literature for LAB [1,29]. One up-regulated protein was identified as equivalent to p40/Msp2. Given the increasing number of available genome sequences in LAB, and the Bacteria more generally, and improvements in bioinformatic tools for protein analysis over the last few years, we revisited the broader phylogenetic relationships between this and another structurally-similar PG hydrolases in this strain. The two related but distinct enzymes, which share a common CwlO domain, which is commonly found in PG hydrolases, and similar domain layout, are divergently related to proteins occurring broadly in the *Firmicutes* and their bacteriophages, prophages or plasmids, with the p40/Msp2 homologue showing modelled structural similarities to *Streptococcus* homologues (SibA-CHAP, SagA and PcsB proteins). The CHAP-CwlO protein was only detected in a limited number of *Lactobacillus* species. The PcsB protein has been proposed as a candidate for new pneumococcal vaccine development, given that it is essential for normal cell growth and is highly conserved in this genus [30]. Understanding the relationship between the structural similarities between conserved homologues in other genera, such as the probiotic *Lactobacillus* species, will inform the discussion on this matter.

## 2. Results

### 2.1. Confirmation of Speciation

All strains were confirmed as *Lactobacillus* species using PCR primers described by Dubernet et al. [31,32]. GCRL 46 was originally classified as *L. paracasei* [32] based on rRNA VI region sequencing. However, while intergenic spacer region sequencing can differentiate between *L. rhamnosus* and other *L. casei* group members [33] differentiation between *L. casei* and *L. paracasei* using similar approaches is problematic [34]. Recent large-scale analysis of genes in the core genome of this group indicated that the type strain for *L. casei*, ATCC 393, plus a small group of genetically-related strains, constitute a clade which the authors recommend remain designated as *L. casei* [35]. Other strains not within this clade, which are historically named *L. casei* and *L. paracasei*, form a distinct, separate clade (A) and it was recommended that all of these strains be renamed *L. paracasei*. Despite the majority of strains in this clade demonstrating >98% average nucleotide identity (ANI) similarity to each other, and to the *L. paracasei* type strain ATCC 25302 and ATCC 334 (from our ANI analysis of all *L. casei* and *L. paracasei* genomes available in the IMG database), the issue of nomenclature remains unresolved. In the present study, we use the names *L. casei* and *paracasei* synonymously and use the species names that have occurred in prior literature for consistency; broader in silico analysis against published genomes of the *L. casei* group members classified GCRL 46 in clade A and we refer to this strain as *L. paracasei* in the text.

### 2.2. Acid Resistance

**Figure 1:**
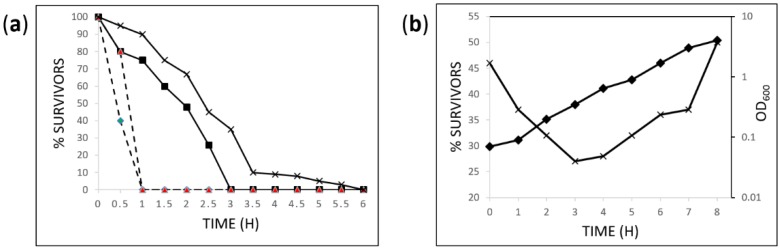
Acid resistance and impact of growth phase on resistance. Panel (**a**): overnight MRS cultures of *L. acidophilus* ATCC 4356 (♦ and ■) and *L. paracasei* GCRL 46 (▲ and X) were diluted into MRS at pH 2 (dashed lines) or 2.5 (solid lines) and viable counts determined every 30 min to determine % survivors relative to time zero. Panel (**b**): strain GCRL 46 was cultured in fermenters without pH control, growth monitored by OD_600_ (♦) and samples taken after inoculation then hourly to determine % survivors (X) after 2 h in MRS broth at pH 2.5.

Previous characterization of *L. paracasei* GCRL 46 in shake-flask culture using buffered media indicated the optimal pH for growth was 6.5 [36]: the maximum specific growth rate in fermenters indicated a similar optimum at pH 6.3 (Figure S1) and growth kinetics were determined for culture at pH 4.0 and uncontrolled pH conditions (Figure S2 shows an example for growth at optimal pH). *L. acidophilus* ATCC 4356 was selected to benchmark acid resistance of *L. paracasei* GCRL 46 as this strain has been reported as acid tolerant and showed greater resistance than *L. casei* strains tested in parallel [37]. Figure 1a shows that the % cell survivors declined rapidly at pH 2 after 0.5 h exposure for ATCC 4356 and viable cells were not detected within 1 h for both ATCC 4356 and GCRL 46. At pH 2.5, clear differences in rate of decline in viability were seen, where GCRL 46 retained higher cell viability over a longer period relative to ATCC 4356, indicating greater tolerance to acidity (Figure 1b). When cells from different growth phases of GCRL 46 were assessed for acid resistance, cells from mid-exponential growth were more acid sensitive than cells from earlier and later stages of culture. This is consistent with stationary phase cells entering a general stress state which results in stress cross-protection, as shown in *L. plantarum* [38].

### 2.3. Growth at Low pH Induces Synthesis of Cell Surface Proteins

**Figure 2:**
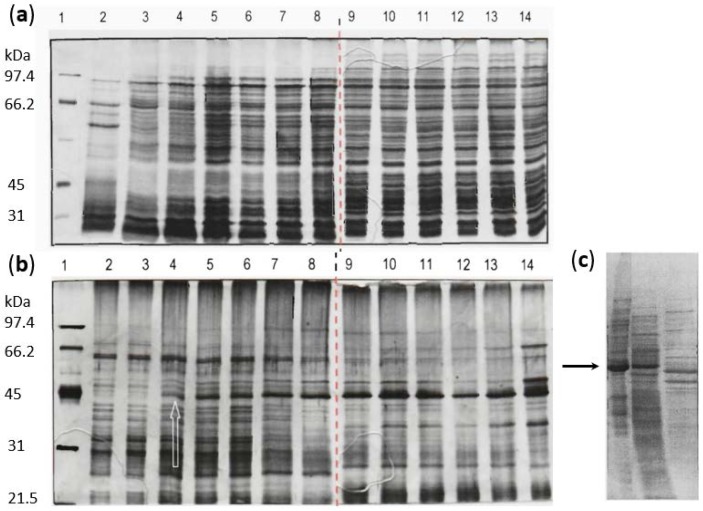
SDS-PAGE gel images for GCRL 46 cells cultured at pH 6.3 or 4.0. Cells were cultured anaerobically in fermenters at set pH of pH 6.3 (panel **a**) and 4.0 (panel **b**), sampling hourly from inoculation (lanes 2–13) and at 24 h (lanes 14) for analysis of whole cell preparations. Size markers are shown in lane 1. The red line shows where parallel gels have been joined to visualize these data; the white arrow indicates initial up-regulation of a protein band of approximately 42 kDa. Panel (**c**) shows (left to right) whole cells, cell debris after centrifugation and the cytosolic fractions for cells culture at pH 4.0 for 24 h. The black arrow points to the ca. 42 kDa protein band.

*L. paracasei* GCRL 46 was harvested hourly from fermenters for cultures with set pH of 6.3 and 4.0 and proteins in whole cell preparations separated on SDS-PAGE (Figure 2a,b). Comparing the temporal expression of proteins under the two growth conditions showed that a major, broad band in the molecular weight (MW) range 40–45 kDa became increasingly dominant across the growth cycle at pH 4.0, which is not apparent in the parallel series at 6.3. Other proteins in the MW range 30-50 kDa were also relatively up-regulated at pH 4.0 during late exponential and at stationary phases but were comparatively minor proteins. The relative intensity of protein bands was determined by scanning gel images using UN-SCAN-IT gel imaging software for the up-regulated protein band at pH 4.0 and for the multiple protein bands in the equivalent region (MW 38–45 kDa) for extracts of cells cultured at pH 6.3 (Figure S3). At pH 6.3, proteins in this MW range did increase across the growth cycle but to a lesser extent than at pH 4.0 and several minor proteins (% density relative to total) of similar MW were observed.

Time-course experiments were also performed using different culture conditions, including maintaining pH at 6.3 until late exponential growth then not controlling the pH to stationary phase; growing cultures to exponential phase then shifting to pH 4.0; or not controlling the pH throughout the growth cycle (Figure S4): in all of these cases, greater synthesis of proteins in the MW range 40–45 kDa was observed, similar to the control at pH 4.0 run in parallel. Spiking pH from 6.3 to 4.0, then returning to pH 6.3, did not produce similar changes in protein profiles in this size range, confirming that up-regulation of protein expression was linked to growth at low pH and likely also linked to protein synthesis late in the growth cycle (see Figure S4).

Stationary phase cells of GCRL 46 were subsequently lysed by bead beating and fractionated by centrifugation to remove cell debris plus unbroken cells from the released proteins (cytosolic fraction). The corresponding whole cells, cell debris and the cytosolic fractions were compared on SDS-PAGE (Figure 2c). Although a major band in the 40–50 kDa was seen in whole cells and the cell debris fraction, the same protein band was not dominant in the cytosolic fraction. This indicated that the proteins in that band were removed during centrifugation and suggested that the proteins were bound in surface structures pelleted during centrifugation.

Given that analysis of the cytosolic fraction would not have been useful in exploring the nature of the surface proteins upregulated by growth at low pH, proteins in whole cell lysates were separated by preparative SDS-PAGE and N-terminal protein sequencing performed. Although it was likely that the broad protein band contained several proteins, a clear N-terminal sequence was obtained, with only the amino acid in the eleventh position reported as equivocal: DTSDSIASNK(S/Q/D)ETNALLKQI.

### 2.4. Characterization of the 42 kDa Acid Up-regulated Protein as a Peptidoglycan d,l-endopeptidase

**Figure 3:**
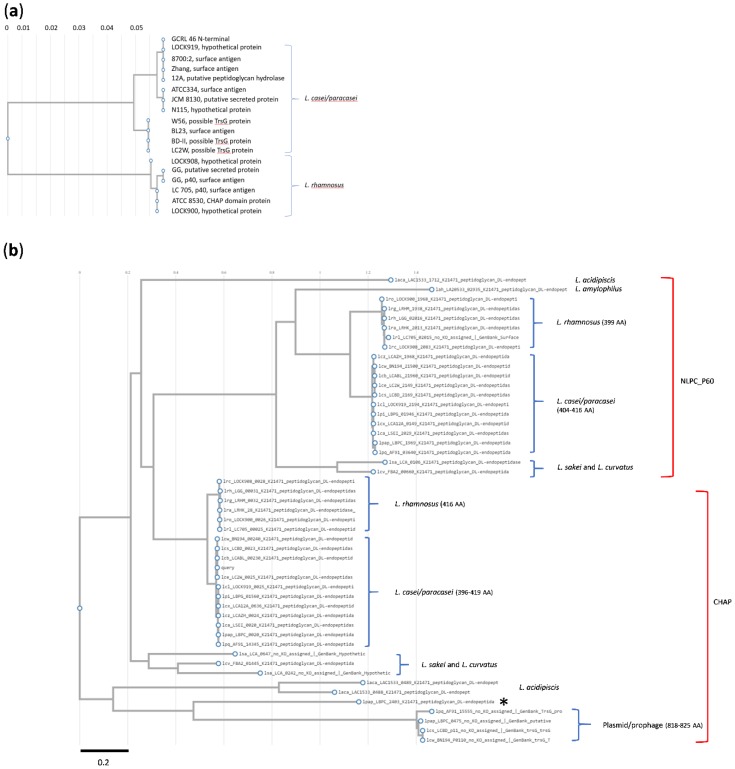
Mid-point rooted phylograms constructed using KEGG TREE for *Lactobacillus* peptidoglycan d,l-endopeptidases. Panel (**a**): the query sequence in BLASTp searches was the 20 amino-acid N-terminal sequence for the acid up-regulated protein from *L. paracasei* GCRL 46. Panel (**b**): the query sequence was the protein identified from searching the genome of *L. casei* GCRL 163 using the N-terminal sequence; other genera in this list (three examples, *Enterococcus* and *Vagococcus* species) were excluded. The red brackets indicate the protein groups sharing common domains, CHAP or NLPC_P60 amidase. The size ranges of proteins (number of amino acids, AA) seen in closely-related clades are shown in parentheses; * notes a protein of 325 AA in *L. paracasei* subsp. *paracasei* JCM 8130 (LBPC_2403) that is likely phage-related.

The 20 amino acid (AA) N-terminal sequence was subjected to BLASTp in KEGG, using all three possible amino acids in the eleventh position: matched protein sequences were only detected when serine was in this position, with 17 hits in *L. casei* group species where the proteins were variously annotated as hypothetical (secreted) proteins, peptidoglycan hydrolases, surface antigens, possible TrsG proteins and analogues of the p40 surface antigen or secreted protein of *L. rhamnosus* (Figure 3a). The corresponding N-terminal regions in these proteins were closely related (branch lengths in the mid-point rooted phylogram of 0.05–0.06) and two clades of proteins were observed, corresponding to *L. rhamnosus* and *L. casei*/*L. paracasei* strains.

The N-terminal sequence was used to search the genome of *L. casei* strains GCRL 163 and MJA 12: a unique protein was detected in both genomes and this sequence was used in BLASTp to explore alignment with other *Lactobacillus* proteins. The phylogram for *Lactobacillus* species (Figure 3b) again showed two, closely-related clades for proteins which contained a CHAP (cysteine, histidine-dependent amidohydrolases and peptidases) domain (Figure 3b). Within the clade containing *L. casei* and *L. paracasei* strains, alignment scores were >95% and protein size varied between 396 AA and 419 AA. Multiple sequence alignment for proteins in this clade (see Figure S5 for representative sequences) indicated that most proteins contained a 28 AA signal sequence upstream from the N-terminal sequence detected in GCRL 46, with the exception of the largest protein in this group, specified by gene locus BN194_00240 (*L. casei* W56, 419 AA), where this was 36 AA. Other single amino acid substitutions were noted in the signal peptide, N-terminal and catalytic domain sequences, in addition to deletions and substitutions which accounted for MW differences.

The CHAP-domain proteins (416 AA) in the second, closely-related clade of *L. rhamnosus* strains showed >88% sequence identity with the GCRL 163 protein. Phylogenetically the nearest neighbors to these CHAP-domain proteins were *L. sakei* and *L. curvatus* species (384–412 AA), although the alignment scores were 46–50%, indicating sequence divergence. The CHAP domain (pfam identifier PF05257) is commonly found in amidases, including peptidoglycan hydrolases, and is a member of the NLPC_P60 superfamily [39]. KEGG gene orthology indicated that these proteins, and many of those shown in Figure 3, are peptidoglycan d,l-endopeptidases, based on the presence of the CwlO motif (K21471). The CwlO motif is the only member of the cl25603 superfamily described as the uncharacterized N-terminal domain of peptidoglycan hydrolases: peptidoglycan lytic activity associated with the presence of this motif in proteins was originally described in *Bacillus subtilis* [40].

### 2.5. Phylogenetic Relatedness to Other Endopeptidases in Lactobacillus Species and Plasmid/Prophage Proteins

**Figure 4:**
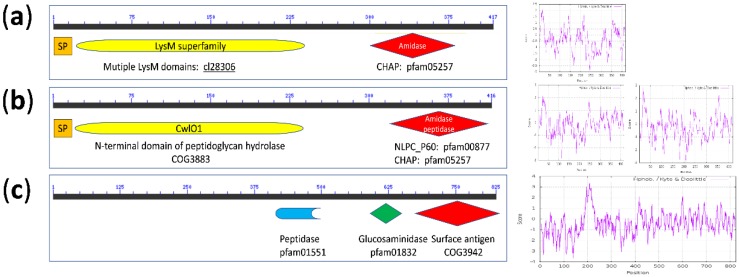
Domain layout and hydrophobicity profiles of peptidoglycan d,l-endopeptidases. Domain names and identifiers indicated are from NCBI CDD; if detected, signal peptide region (SP) are shown. Panel (**a**): *L. acidipiscus* LysM-CHAP-domain glucan-binding protein (gene locus Lac1533_0489). Panel (**b**): *L. casei* group proteins with either a NLPC_P60 or a CHAP C-terminal domain (probable TrsG protein), with CwlO domains (specified by gene loci BN194_21500 and BN194_00240, respectively, with Kyte-Doolittle plots in this order). Panel (**c**): plasmid/bacteriophage TrsG protein (gene locus BN194_P0110); the surface antigen domain (COG3942) is identified as a CHAP domain in KEGG (pfam 05257.16) and UniProt. Hydrophobicity scores >1.9 indicate likely transmembrane regions.

Figure 3 further showed a second group of proteins with NLPC-P60 domains plus the CwlO motif: the *L. rhamnosus* proteins were distinct from the *L. casei*/*L. paracasei* proteins (73% alignment score) so two clades were observed again. The *L. casei* NLPC_P60 proteins showed an alignment score of 32.6% with the CHAP hydrolases (Figure S6 shows examples of sequence alignment for selected *L. casei* strains), indicating some sequence similarity in the CwlO motif but divergence in the endopeptidase domain region, despite the CHAP domain being in the NLPC_P60 superfamily. Plasmid and bacteriophage proteins (818–825 AA) were distinguishable from the smaller CHAP- and NLPC_P60-domain endopeptidases, although they contained domains annotated as surface antigen or CHAP (Figure 4), whether they were on plasmids or integrated into the genomes as prophage or in regions with bacteriophage remnants (e.g., LBPC_0475, GC content 46.8%). One exception was the 325 AA protein in *L. paracasei* subsp. *paracasei* JCM 8130, gene locus LBPC_2403, which is described in UniProt as an uncharacterized protein with CHAP-SibA-like domains. Figure S7 provides the layout of genes in strain JCM 8130 around this locus and compares this with prophage and plasmid genome maps. The latter are typified by the presence of genes associated with integration, phage capsid production and packaging, plasmid/phage replication/plasmid copy number, type IV secretion system (VirD4, TraK, K03205) and transposases associated with specific insertion elements (IS). The genome region around LCPC_2403 contains several small, uncharacterized proteins in addition to type IV secretion system (VirD4), TrwB, TraG_C, integrases and transposases, suggesting plasmid or bacteriophage origin. The DNA GC content of the gene is 51.3%, which is higher than the average seen for genomes of *L. casei* group species (46.6%) [41], also suggestive of lateral and relatively recent acquisition of this endopeptidase in this strain’s genome. The GC content of genes encoding the CHAP- (BN194_00240) and NLPC_P60- (BN194_21500) domain proteins is 48% and 46.5% respectively, suggesting that if these genes were of plasmid or bacteriophage origin, acquisition was not recent. Genome maps (Figure S7) indicated that the genes for both proteins are not within genome islands rich in plasmid- or prophage-associated genes, but BN194_00240 is close to the origin of replication and ‘downstream’ of prophage genes at the end of the circular genome. This genome layout is detected in several strains of the *L. casei* group, including GCRL 163, but not in all strains [41], and exemplifies the diversity in genome makeup in *L. casei* group strains. Interestingly, strain JCM 8130 contained three genes for CHAP-domain proteins, LBPC_0020 (CwlO-CHAP, p40 homologue), LBPC_0475 (825 AA, phage), LBPC_2403 (325AA, transglycosylase-CHAP) as well as the gene specifying the CwlO-NLPC_P60 (LBPC_1969) protein.

Although the degree of sequence identity was low, the overall domain architecture of the *L. casei* group endopeptidases was similar (Figure 4b), consisting of a signal peptide region (SP), N-terminal CwlO domain and a C-terminal catalytic region (CHAP or NLPC_P60). Hydrophobicity plots indicated likely transmembrane regions, also shown from the multiple coiled-coil regions noted in UniProt. Indeed, the NCBI conserved domain database (CDD) lists 1036 similar architectures with 4442 non-redundant entries for genes specifying SagA-like proteins (surface antigen proteins detected in pathogenic group A streptococci species as a secreted, immunoglobulin-binding—SibA, also named PcsB proteins) [42], which have structures similar to Figure 4b, dominating the Bacteria. This indicates broad conservation of this architecture, particularly in the *Firmicutes* (currently 2753 entries).

A CHAP-domain, glucan-binding protein in *L. acidipiscus*, however, showed a different architecture: a region containing multiple LysM motifs (approximately 40 AA globular domain) followed the SP, with a conserved C-terminal CHAP domain, with lower hydrophobicity in the coding region beyond the signal sequence. In contrast, the architecture of the 825 AA bacteriophage protein lacked a SP and showed hydrophobic regions around AA 175–225 (noted as a periplasmic-specific motif in CDD searches) and across the three catalytic domains detected, indicating a transmembrane location but unlikely secretion.

### 2.6. The Two *L. casei* Group Peptidoglycan d,l-endopeptidases Show Different Broader Phylogeny

**Figure 5:**
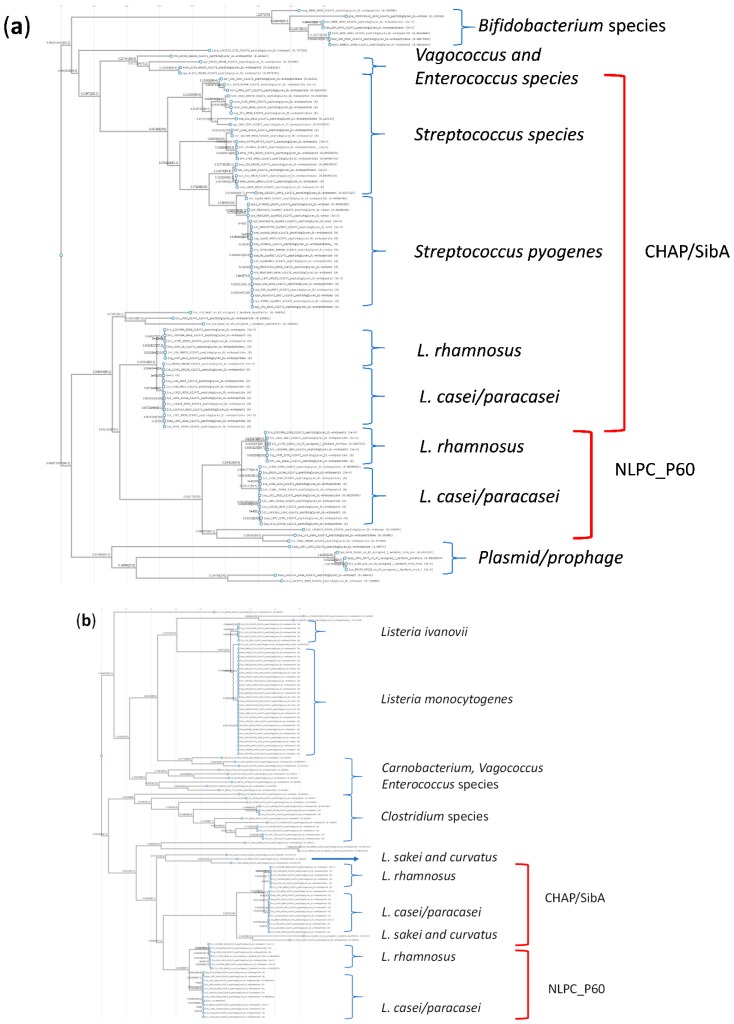
Mid-point rooted phylograms for top 100 aligned proteins for (**a**) query sequence of CHAP/SibA-domain protein (equivalent to gene locus BN194_00240) and (**b**) query sequence NLPC_P60 domain (equivalent to gene locus BN194_21500) protein from *L. casei* GCRL 163.

BLASTp searches using the sequences for the CHAP and NLPC_P60 proteins from the genome of *L. casei* GCRL 163 as queries are shown in Figure 5. Mid-point-rooted phylograms for the top 100 hits indicated that, despite the low sequence alignment scores (<28%), the CHAP protein was most closely related to *Streptococcus pyogenes* and other *Streptococcus* species proteins, whereas for the NLPC_P60 protein was more closely related to similar proteins in *Clostridium* and *Listeria* species. When phylograms extended to the top 500 hits (Figure S8), the CHAP endopeptidase demonstrated relatedness to *S. mutans* and *S. pneumoniae* CHAP/SibA proteins, whereas *Staphylococcus epidermidis* proteins detected as related contained only the CHAP domain (not the CwlO domain) and *S. aureus* domain structure was LysM-CHAP, similar to the *L. acidipiscus* domain architecture in Figure 4 but in smaller proteins (265 AA). Interestingly, the phylograms (and a name search in UniProt) failed to detect the CHAP domain in several *Lactobacillus* species, including *L. acidophilus*, *L. plantarum*, *L. helveticus*, *L. reuterii*, *L. delbrueckii* and *L. johnsonii*. In contrast, the NLPC_P60 domain, which is more widely spread in the Bacteria and occurs in proteins with a variety of functions and accompanying domains [43], was detected in 2062 entries in *Lactobacillus* species in UniProt, including the *L. acidophilus* group. In the top 500 hits for this protein in a KEGG search using the full sequence of the protein, other genera detected included *Streptomyces*, *Bacillus*, *Enterococcus* but not *Streptococcus* species: while this observation may be explained by low sequence similarity and annotation failure, it suggests that the spread of genes, or their composite domains, through genera and species was different for the two cysteine peptidases.

### 2.7. The Predicted Protein Structural Models Differentiate between the Two Endopeptidase Types in *L. casei* Group Strains

**Figure 6:**
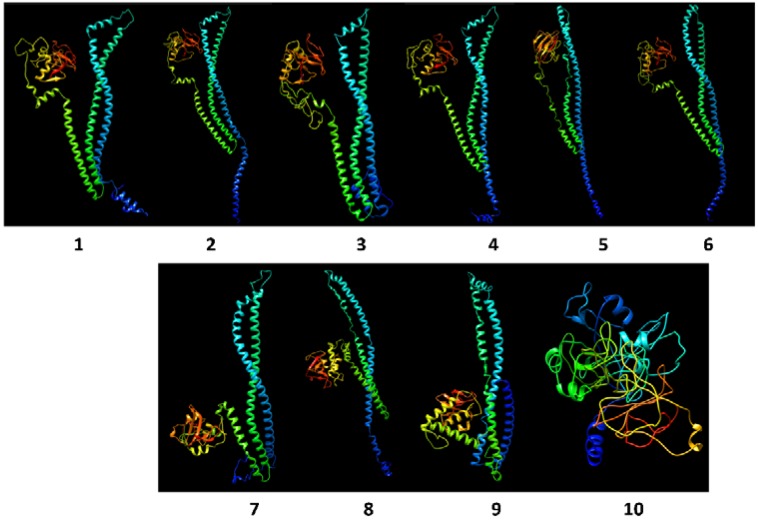
Proteins were modelled using the FASTA sequences available on UniProt or KEGG databases and the annotated genome of strain GCRL163. The upper group (1–6) are for proteins with the CwlO-CHAP domain architecture shown in Figure 4b: 1 = *L. casei* GCRL 163 genome sequence, uncharacterized protein; 2 = *L. casei* W56 possible TrsG protein (BN194_00240); 3 = *S. mutans* SagA protein (Q8DWM3); 4 = *S. pyogenes* SibA secreted protein (Q1JJ91); 5 = *L. rhamnosus* p40 surface antigen (LGG_00031); 6 = *L. rhamnosus* multi-species CHAP-domain containing protein WP_064517214.1. Models 7–9 are for proteins with CwlO-NLPC_P60 architecture: 7 = *L. casei* GCRL 163 genome sequence, putative peptidoglycan hydrolase; 8 = *L. casei* W56 surface antigen (BN194_21500); 9 = *L. rhamnosus* surface antigen (LRHMDP2_482). Model 10: *L. acidipiscus* LysM-CHAP architecture, glucan-binding protein (LAC1533_0489). Protein models are not to scale. Ribbon colours show blue at the N-terminal to red at the C-terminal.

Figure 6 shows a collage of proteins modelled using the Phyre2 platform; Figure S9 shows predicted metal ion binding sites for selected proteins. The crystalline structure of the *S. pneumoniae* CwlO-CHAP protein, called PcsB (named from its proposed role as a protein required for cell wall separation in group B streptococci), has been reported [30]. PcsB proteins have been characterized in *S. mutans* (GbpB) and *S. agalactiae* and homologous proteins occur in all *Streptococcus* and *Lactococcus* species (see ref. [30] and Figure S8). The crystalline structure is described as consisting of four features: a signal peptide, a coiled-coil domain (CwlO), a linker region and the catalytic domain at the C-terminus. The template for predicting the structures in the *Lactobacillus* proteins included the *S. pneumoniae* PcsB crystalline structure and hydrolases from multiple species (including *Mycobacterium tuberculosis* invasin protein, peptidase M23 and cysteine proteinases in the NLPC_P60 family), all with >99% confidence (probability that the sequence and template are homologous) for regions aligned with templates. Models for the *L. casei* CwlO-CHAP proteins were highly similar to the *S. mutans* and *S. pyogenes* proteins. However, the *L. rhamnosus* homologues showed variability in predicted architecture, despite multiple modelling over an extended period–often producing models with the coiled-coil regions bunched. Figure 6 shows a proposed structure for the p40 protein (sequence from KEGG) and the multi-species CHAP-domain protein of *L. rhamnosus* (sequence from NCBI), where the latter shows the four features seen in streptococci structures but was distinct from the p40 model: the AA sequence differed by three AAs between the p40 protein in LGG and the multi-species CHAP-domain protein sequence, one in the signal sequence and two in the catalytic domain, so differences in the models cannot necessarily be explained by minor sequence differences. N-terminal differences in the models are in line with the observed size differences seen for the signal peptide (Figures S5 and S6).

Although the general architecture of the modelled NLPC_P60 proteins showed features similar to the *S. pneumoniae* template (PDB code 4CGK), the key difference was the relative position of the catalytic region. Attempted modelling of another protein with an NLPC_P60 domain detected in the genome of *L. casei* (equivalent to the protein specified by gene locus BN194_02820 in strain W56, and protein p75 of *L. rhamnosus* LGG) was not successful, as 54% of the sequence was predicted as disordered. The LysM-CHAP protein detected in *L. acidipiscus*, which lacked the CwlO motif, showed a catalytic region (green structure in model 10) but no parallel coiled regions, consistent with the domains detected.

All of the proteins had high-confidence predicted binding sites for Zn and some for Mg, usually in the coiled regions; the NLPC_60 proteins had predicted metal binding also in the catalytic regions.

## 3. Discussion

How LAB respond to stressors has been the subject of increasing research at the proteomic and transcriptomic level over the last 20 years, due to the importance of understanding the underlying mechanisms of coping with potentially-lethal or growth-limiting assaults that are encountered during passage through the GIT or during food fermentations [1,29]. The functional performance of fermented foods (acceptable organoleptic features and extent of shelf-life during storage) and probiotics depends on the continued viability of LAB in their environment, particularly to meet the FAO/WHO requirements as “live microorganisms which when administered in adequate amounts confer a health benefit on the host” [44]. Consequently, evaluating strain fitness for purpose has become increasingly important for economic and validation of health claim reasons, particularly with expanding markets for consumption of probiotics as dietary adjuncts [45]. Acid and bile stress responses are amongst the most well documented in LAB, given that survival during passage through the stomach is required for probiotic bacteria to exert their benefits lower in the GIT. Responses commonly detected in multiple species include alteration to the cytoplasmic membrane, where the fatty acid composition of phospholipids changes to impact membrane rigidity and permeability [1,29,46,47,48]. Despite the accumulating knowledge regarding the nature of cell wall hydrolases in the *Firmicutes*, particularly in *Streptococcus* species [30], and their functional role in probiotic *Lactobacillus* species [25,26,27,49,50], there is little documented on the regulation of this class of proteins in *Lactobacillus* or other LAB in terms of protein synthesis during exposure to stress [1,29,51]. Of growing interest is the make-up of LAB cell surfaces and the complement of proteins, including PG hydrolases, which are exposed to or occur in the external environment, as many of these components will impact on interactions with host cells to deliver health benefits [15,52]. In silico analysis of LAB genomes based on sequence homology, protein-domain searches and detection of motifs found in secreted, transmembrane and anchored proteins showed that the *L. casei* has one of the highest numbers of predicted surface and secreted proteins in the genus *Lactobacillus* [15,52]. However, many of the proteins are functionally uncharacterized so their potential roles at the surface can only be predicted from detection of conserved domains. Vollmer et al. [19] noted that assigning specific functions to PG hydrolases is often difficult, due to the presence of multiple enzymes performing similar functions relating to PG turnover (redundancy) and domains found in PG hydrolases occur in enzymes with other catalytic activity, as noted for NLPC-60 and CHAP domains which occur in presumptive PG hydrolases and are widespread in other enzymes [39,43]. Further challenges in predicting enzyme function arise from computational analysis leading to the misannotation of protein sequences which are not experimentally characterized [53] and multiple abbreviated names used in the literature for the same activity, as observed for the SibA/CHAP protein in *Streptococcus* species. In the present study, several proteins were observed as up-regulated when *L. paracasei* GCRL 46 was cultured in fermenters at low pH, or when the pH of the medium was decreased to 4.0 and sustained at this pH during culture, but similar changes were not detected when cells were temporarily shifted to low pH then returned to optimum (acid shock). An up-regulated, surface-located protein detected by N-terminal sequence analysis was variously annotated as a surface antigen, uncharacterized, possible hydrolase and TrsG protein in *L. casei* group species, *L. casei*, *L. paracasei* and *L. rhamnosus*, although the sequence identity between the *L. casei*/*paracasei* and *L. rhamnosus* clades was 88%. TrsG was originally detected in *S. aureus* plasmids as part of the transfer machinery [54] and contains a CHAP domain (UniProt), hence the annotation of the ca. 42 kDa upregulated protein as TrsG. Sequence similarity determined this protein to be a homologue of p40 (Msp2) (equivalent to the protein specified by gene locus BN194_00240 in *L. casei* W56), which had previously been reported as one of the major secreted proteins in probiotic *L. casei* and *L. rhamnosus* strains [25,26,27]. Although the other major secreted protein in the *L. casei* group, p75/Msp1, was characterized as a PG hydrolase by degradation of muropeptides [25], the role of p40/Msp2 in PG turnover, septation and daughter cell separation has not been fully determined in *L. casei* group species: knock-out mutants either failed to cause phenotypic changes in *L. casei*, due to redundancy, or were not generated in *L. rhamnosus* [25,26,27]. Furthermore, while Bäuerl et al. [27] could demonstrate some degradation of muropeptides by recombinant Msp2 and clear degradation by Msp1, zymogram analyses by others failed to demonstrate activity in extracellular culture fluids of *L. casei* BL23 for Msp2 (which were positive for Msp1) [26] or for recombinant Msp2 from *L. rhamnosus* LGG for gels loaded with 20 µg of purified protein [25], indicating contradictory evidence for the activity of Msp2 as a PG hydrolase. Immunofluorescence localized Msp2 at cell poles during division of *L. rhamnosus* LGG, implicating this protein in PG turnover and daughter cell separation. Only one other PG hydrolase in the *L. casei* group has been functionally characterized as an autolysin from its mureinolytic activity [55], which is distinct from Msp1 and Msp2. Although transcriptomic studies indicated that both Msp1 and Msp2 are expressed during logarithmic growth [26], there are no other reports on the impact of environmental conditions on protein expression levels. We believe that the current work is the first report of up-regulation of a surface-located, hydrophobic, Zn-binding presumptive PG hydrolase, Msp2, in response to prolonged acid stress in an *L. casei* group species.

Many of the features of the p40/Msp2 protein in the current report were originally described by Bäuerl et al. [27], who identified the presence of CHAP and CwlO (COG3883) domains, low sequence identity with similar proteins in *Bacillus*, *Clostridium*, *Streptococcus* and *Listeria*, and a signal peptide. Furthermore, these authors demonstrated that both p40 and p75 presumptive hydrolases had a surface and extracellular location by extraction of surface proteins with 1.5 M LiCl and detection by Western blotting using immune serum prepared against p40 and p75 (noting that the p40 antiserum cross-reacted with the p75 protein, which was also noted previously [23]). These authors also failed to detect both proteins in the cytosolic fraction, as observed in the current report for the control cultured at pH 6.3, nor in a cell envelope (cell membrane/wall) fraction despite observing several other cross-reacting proteins. We suggest that the cross-reactivity is due to the presence of NLPC_P60 superfamily domains which are in both p40 and p75, despite low sequence similarity between these proteins including the C-terminal region (29.3%), and the other proteins detected in Western blots. Bäuerl et al. [27] concluded that p40 occurred exclusively in the *L. casei* group of bacteria. However, we were able to show broader phylogenetic relationships for the p40 homologue in *L. paracasei* GCRL 46 and detected a structurally-related presumptive PG hydrolase which contains an N-terminal CwlO with a C-terminal NLPC_60 domain (specified by gene locus BN194_21500) with 34% similarity to the CHAP protein. Although the sequence similarity was <50%, p40 homologues were detected in a limited number of *Lactobacillus* species, including the most closely related sequence in strains of *L. sakei* and *L. curvatus* for proteins with the same overall domain layout and structural architecture as p40, indicating that p40 is not unique to the *L. casei* group. However, p40 homologues were not detected in the *L. acidophilus* group species although NLPC_P60-domain proteins were, as reported previously following in silico analysis of published genomes of *L. acidophilus* for detecting presumptive autolysins [49]. The *L. casei/paracasei* p40 homologue was phylogenetically most closely related to *Streptococcus* PcsB (Gbp, SibA/CHAP) proteins, whereas the CwlO-NLPC_P60 protein was more similar to *Clostridium*, *Listeria* and (with less similarity) *Bacillus* homologues. The difference in analysis by Bäuerl et al. [27] and the current study arises from the greater variety of *Lactobacillus* and other genomes now sequenced and the differentiation between detecting the common CwlO domain in structures that lack the CHAP domain but may contain the NLPC-P60 domain. There are no published studies on the role of the CwlO-NLPC_P60 protein in *L. casei* group strains, including its possible role in PG turnover. The modelled structure of this protein showed similarities to the p40 protein which suggests putative PG hydrolase activity: the question of whether this enzyme, or other uncharacterized PG hydrolases, complement p40 activity so that inactivation of p40 would result in no phenotypic changes remains a matter for further exploration through transcriptomics, proteomics and physiological studies.

The CHAP domain was also detected in proteins lacking the CwlO domain, but containing other domains including LysM in modular structures, particularly in prophage-related proteins with presumptive activities related to modification of the cell wall but lacking signal peptides. Bacteriophage endolysins are often large proteins with multiple modular domains, including CHAP, which occur in phage that infect or reside in *Lactobacillus*, *Streptococcus*, *Staphylococcus*, *Listeria* and other genera [56]. It is likely that acquisition, and loss, of domains through exchange with phage and plasmid genes has contributed to the current profile of putative PG hydrolases in *L. casei* group species, given the distinct phylogeny of the two classes of CwlO-containing proteins. This is supported by our observation for strain *L. paracasei* subsp. *paracasei* JCM 8130, which contained multiple genes encoding proteins with CHAP domains, including one with GC content indicative of recent acquisition but with high sequence similarity to the p40 CHAP domain detected in this study (e-value = 4 × 10^−45^). It is likely that genes are exchanged regularly between phage and plasmids, as predicted in *Bacillus* species [57], but ancient acquisition of the p40 homologue in *L. casei* is indicated from GC content similar to the average for the genome. The genomes of *L. casei/paracasei* consist of a core and variable regions which make up the pan-genome, indicative of a species adapted to specialized environments [41,58]. Furthermore, there is evidence that the genome of *L. rhamnosus* LGG can modify during product storage indicating genetic rearrangements in genome islands containing insertion elements [59]. Understanding the phylogenetic origins of the domains occurring in PG hydrolases and the fluidity of their passage across genera may identify the key enzymes associated with PG synthesis and turnover in *Lactobacillus* species and how probiotic bacteria exert their beneficial effects, including pathogen exclusion in species with a similar genetic makeup and PG hydrolase complement.

The regulation of PG hydrolases in cells involves a crucial balance between transcription and post-translational control of enzyme activity, to provide sufficient expression of protein synthesis for PG turnover and cell separation without exerting the potentially lethal process of autolysis [19,57]. Regulation of expression of PG hydrolases has been extensively studied in *B. subtilis*, *S. aureus* and *S. pneumoniae*: the WalKR regulon includes PG hydrolases involved in cell separation, such as PcsB in *S. pneumoniae* (see ref 30). Although the PcsB protein is well characterized and contains the putative catalytic domains linked with PG hydrolase activity (CwlO-CHAP), demonstrating hydrolytic activity has not been successful [30]. The model proposed to explain this phenomenon suggests post-translational control of activity through interaction between PcsB and the surface-located FtsEX complex, where the physical architecture of the PcsB protein and molecular distances obtained for the crystallized PcsB protein support this proposed interaction. In this model, PcsB is bound to the FtsEX complex and is normally inactive at the cell surface. As cell division occurs and septum formation progresses, two PcsB-FtsEX complexes are brought together across the separating cell membrane interfaces forming a dimer of the complexes, activating the degradation of PG and separating daughter cells. Bartual et al. [30] further propose that the highly-abundant PcsB protein acts locally and is shed into the extracellular medium once cell separation is achieved, hence detection of the hydrolases as secreted proteins in streptococci. Despite relatively low sequence similarity between the PcsB and p40/Msp2 proteins, they are phylogenetically related and occur in a different lineage to the more widely-spread NLPC_P60-domain proteins. Given that the p40/Msp2 protein is phylogenetically related to the PcsB protein, and the modelled structures in *L. casei* group strains showed high similarity with the *Streptococcus* PcsB/SibA/CHAP proteins PcsB, it is likely that members of this family of proteins will behave similarly in *L. casei* group and other low GC Gram positive species where the architecture of the proteins is conserved. The *L. casei* group p40 protein has not been crystallized nor has the mechanism of its regulation been explored, but it is likely that its location and regulation would be similar to *Streptococcus*. Accumulation of this protein at the cell surface during prolonged acid stress in GCRL 46 may suggest altered transcriptional regulation of expression and that p40 activity is insufficient to achieve cell separation alone. The CwlO-NLPC_P60 showed a similar, but distinct, architecture where the hydrolase ‘head’ was not in the same position relative to the coiled regions of the PcsB and p40 homologue. This may be an artefact of modelling which would be clarified by protein crystallization and analysis of the atomic distances to determine whether the FtsEX model would apply to this enzyme also. The SagA/PcsB proteins are considered targets for vaccine or new antimicrobial development, given the essential nature of this protein in pathogenic streptococci [30]: vaccines and antimicrobials based on this structure would need to consider the impact on commensal, non-pathogenic species that reside in similar mucosal environments.

Our prior work with GCRL 46 showed that LiCl-extractable proteins were altered following growth at low pH [60]. The p40 protein was not seen as a major component of the LiCl extracts or extracellular medium [61], but the growth conditions, point of harvest, use of buffers to control pH (introducing osmotic pressure differences to the current report) rather than fermenters may account for these observations. LiCl extraction has been used to discover new putative PG hydrolases and other surface proteins in *L. acidophilus* [49,50,62]: this species has an S-layer which is implicated in forming a scaffold for PG hydrolases and other surface proteins, given co-extraction with the S-layer. However, several *Lactobacillus* species, including *L. casei* group, lack an S-layer and the concentration of proteins in the extracellular medium and at the surface appears to be low [49,63], hence the need to detect proteins by Western blotting when examining the p40 homologue in *L. casei* [27]. Failure to detect up-regulation of presumptive PG hydrolases in *L. casei* in prior reports [47,48] is reasonably explained by growth conditions and methodologies used, as the normal approaches have employed acid shock rather than prolonged stress throughout culture at low pH, annotated functional assignment not identifying presumptive PG hydrolases and strains used. Future work on examining the changes in surface under prolonged stresses, rather than transient expression of genes responsible for immediate protection of cell structures and protein function, may identify a broader suite of proteins needed for continued growth and the recruitment of enzymes to the cell surface for the protection of PG integrity.

## 4. Materials and Methods

### 4.1. Bacterial Strains and Culture Conditions

Strains of *Lactobacillus* were originally obtained as a gift from the Commonwealth Scientific and Industrial Research Organization (CSIRO), Food Science Australia laboratories (now Food Innovation Centre, Werribee, VIC, Australia) or purchased from the American Type Culture Collection (Manassas, VA, USA). Type strains used were: *L. acidophilus* ATCC 4356, *L. casei* ATCC 393 and *L. rhamnosus* ATCC 7469. *L. paracasei* VUP 12006 was initially stored in the Victoria University, Werribee, VIC, Australia, culture collection [32] and was renamed GCRL 46 when re-stored in the University of Melbourne, Gilbert Chandler Research Laboratories, Werribee, VIC, Australia [32]. *L. casei* GCRL 163 was originally isolated from maturing Cheddar cheese [64] and its genome was published recently [65]. Strains were cultured routinely using de Man, Rogosa, Sharpe (MRS) broth (Oxoid, Thebarton, SA, Australia) or plates (1.5% agar, Oxoid) and incubated at 37 °C under anaerobic conditions using Oxoid jars with Gas Generating Kit BR038B. Strains were stored in glycerol storage broth (50% glycerol in MRS) under oxygen-free nitrogen at −20 °C as working stocks and −80 °C for permanent stocks or on cryobeads (Protect, Technical Service Consultants Ltd., Lancashire, UK) used according to the manufacturer’s instructions.

### 4.2. rRNA Gene Sequencing for Species Identification

Genomic DNA was extracted from overnight MRS broth cultures of all strains as described previously [32,65] using methods originally detailed in Marmur [66] or using Isolate II Genomic DNA extraction kits (Bioline, Taunton, MA, USA) in accordance with the manufacturer’s directions for Gram-positive bacteria. Several polymerase-chain reaction (PCR) primer sets were used to amplify regions of 16S rRNA genes and 16S-23S rRNA intergenic spacer regions (ISRs): *Lactobacillus* genus-specific primers for ISRs (R16-1/LbLMA1-rev) were described by Dubernet et al. [31]; universal primers 16S-27F and 16S-1492R [67], as modified by Turner [68], were purchased from Sigma Aldrich Pty. Ltd. (Sydney, NSW, Australia); species-specific primers used were ACI16S1/ACI16S2 for *L. acidophilus* [69] and PR1/CAS2 plus PAF/536F for *L. casei* [69,70]. Primers described by Kwon et al. [33] were also used to differentiate between *L. casei* (IDL11F/IDL03R) and *L. rhamnosus* (IDL04F/IDL73R). PCR amplification conditions and amplicon separation techniques are reported in Pepper [32] and Shah [34]. Amplicons were sequenced by Macrogen (Republic of Korea) or the joint Victoria University-Monash University sequencing facility (Monash University, Clayton, VIC, Australia). Sequences were analyzed using the Sequencher software program (Sequenchergenetics version 4.8, Genes Codes Corporation, Ann Arbor, MI, USA) then searched through the Basic Local Alignment Search Tool (BLAST) of the National Centre of Biotechnology Information (NCBI, available online: https://blast.ncbi.nlm.nih.gov/Blast.cgi) or the BLASTn tool in Kyoto Encyclopedia of Genes and Genomes (KEGG, available online: https://www.genome.jp/tools/blast/) to determine the closest matches with known 16S rRNA gene sequences. ClustalW (KEGG BLAST tools) was used for sequence alignment.

### 4.3. Growth in Fermenters and Determination of Acid Resistance

Culture was performed in MRS broth using fully-instrumented, water-jacketed one L Applikon fermenters (Enztech, Woollahra, NSW, Australia), allowing continuous measurement and control of temperature at 37 °C (± 0.5) and pH values (± 0.2). Set pH values in the range of 2 to 8 were achieved using sterile 2 M HCl or NaOH as required prior to inoculation and by automatic addition during culture. Media were autoclaved (30 min, 121 °C) in the vessels and sparged with oxygen-free nitrogen to maintain anaerobic conditions. Oxygen levels were monitored throughout using an oxygen probe and the volume of acid or base dosed during growth recorded. Starter cultures were prepared from frozen stocks by seeding 20 mL MRS broth with two cryobeads or 50 µL glycerol stock culture and incubating 6–8 h at 37 °C before sub-culturing 10 mL of starter culture into 200 mL of fresh medium then incubating overnight at 37 °C without mixing. Cells were collected by centrifugation (10,000× *g*, 10 min), concentrated 10-fold in MRS and vessels seeded aseptically, in duplicate for each test condition, to achieve an initial optical density (600 nm) (OD_600_) of 0.13–0.23 (UltraSpec III UV/Vis spectrophotometer, Amersham Biosciences AB, Uppsala, Sweden). The vessels were stirred at 150 rpm, sparged with nitrogen gas (0.1 L/min), with temperature and pH maintained at set points. In some experiments, the set pH was changed during growth or the pH control not initiated so the pH in the vessels decreased over time. Samples were normally removed hourly for the determination of dry weight, OD_600_, glucose concentration and collection of cells for later protein analysis. If not used immediately, all samples were stored at −21 °C. Growth rates were determined using the Monod equation [71] and specific growth rates between time points, as well as the maximum specific growth rate (µ_max_), calculated automatically using the BASIC program first developed by Viega and Gutierrez [72].

Acid resistance was determined using a method originally described by Gordon and Small [73], replacing the test broth with MRS. Duplicate MRS broths (10 mL) with initial pH adjusted to 2.0 or 2.5 using HCl were inoculated with 50 µL of overnight MRS cultures of *L. acidophilus* ATCC 4356 or *L. paracasei* GCRL 46 and broths incubated at 37 °C. Samples were removed immediately after inoculation then every 30 min for 6 h and again at 24 and 48 h if required. Survivors were enumerated by plating 10-fold serial dilutions onto MRS plates in duplicate and viable counts determined after 48 h at 37 °C. The percentage of survivors was calculated relative to time zero for each pH condition.

To determine the relationship between growth phase and acid resistance, strain GCRL 46 was inoculated into fermenters with an initial pH of 6.3 and cultured without pH control. Samples were removed hourly during growth and 50 µL inoculated into MRS at pH 2.5 then viable counts determined after 2 h at 37 °C, as described above. The averages of percentage survivors were plotted against culture period.

### 4.4. Detection of Proteins by Sodium Dodecyl Sulphate-polyacrylamide Gel Electrophoresis (SDS-PAGE)

One dimensional SDS-PAGE was performed according to the method of Laemmli [74] using vertical slab systems (Modular Mini-PROTEAN^®^ II electrophoresis system and PROTEAN^®^ II XL, BioRad, Gladesville, NSW,Australia; and C.B.S. Scientific Triple Wide System, Spectrum Chemical MFG Corp, New Brunswick, NJ, USA) with 10% or 12% running gels and 4% stacking gel, as described previously [75]. After recording OD_600_, 50 mL culture samples from fermenters were centrifuged, the supernatant removed and cells resuspended in a volume of Tris buffer (0.04 M, pH 7) to concentrate to a standard OD_600_ equivalent to 15. A sample of cell suspension was added to an equal volume of double-strength sample buffer, mixed by vortexing and heated at 100 °C for 5 min, followed by centrifugation (16,000× *g*, 10 min) to remove unbroken cells and debris. Equal volumes of each whole cell extract were loaded onto SDS-PAGE gels when comparing time-course samples. Alternatively, cells were disrupted by bead beating using a tissue homogenizer (MSK Cell Homogeniser, B. Braun, Bella Vista, NSW, Australia): an equal volume of concentrated cell suspension and glass beads (0.10–0.11 mm diameter, Daintree Scientific, St Helens, TAS, Australia) were placed in a stainless-steel homogenizer bottle and treated in 30 s bursts for a total 2 min, with cooling on ice. The homogenate was centrifuged to remove debris (20,000× *g*, 4 °C, 10 min) and the supernatant fluid collected (cytosolic fraction). The cell debris was retained for SDS-PAGE analysis, resuspending in the original sample volume with single-strength sample buffer before heating at 100 °C for 5 min and brief centrifugation (10,000× *g*, 5 min) to pellet beads. Protein concentration in the cytosolic fraction was determined by a modified Lowry assay [76] against a bovine serum albumin standard and equal amounts of protein (1–6 µg) loaded onto gels after heating in double-strength sample buffer. Following electrophoresis, proteins were detected using Coomassie Brilliant Blue R250 or silver staining using standard protocols (http://www.protocol-online.org/, accessed from August 1999 to 10 February 2019) and gels scanned using a Gel-Pro analyzer (Gel-Pro Version 3.0, Media Cybernetics, Rockville, MD, USA) to determine the molecular weight and relative density of the proteins. Gel images were also scanned using UN-SCAN-IT Gel Analysis software 7.1 for relative density (% pixels) and molecular weight estimation (Silk Scientific Inc., Orem, UT, USA).

### 4.5. Protein N-terminal Sequencing

Proteins in whole cell extracts were applied to SDS-PAGE gels in the PROTEAN II XL system and electrophoresed until the 31.5 kDa size standard had reached the bottom of the gel, allowing greater separation of proteins in the size range of 40–50 kDa. Gels were soaked in transfer buffer (10 mM 3-[cyclohexylamino]-l-propanesulfonic acid, 10%, *v/v,* methanol, pH 11.0) for 5 min and electroblotted to polyvinylidene difluoride (PVDF) membranes [77] using a Trans-Blot^®^ Cell (BioRad, Australia) according to the supplier’s instructions. Bands were visualized using Amido Black 10B (0.1%, *w*/*v*, in 10%, *v*/*v*, ethanol, 2%, *v*/*v*, acetic acid, 10 min), small samples of membrane excised and sent to the Australian Proteome Analysis Facility (APAF) (Macquarie University, Sydney, NSW, Australia) for N-terminal sequencing.

### 4.6. Bioinformatic Analyses

BLASTp (KEGG) of the N-terminal protein sequence, using all three combinations of the possible identity of the eleventh amino acid, was used for initial sequence similarity searches. This sequence was also used to search the annotated genomes of *L. casei* strains GCRL 163 and MJA 12 (DDBJ/EMBL/GenBank accession numbers MODT01000000 and MODS01000000) [65] to obtain the full sequence of the protein. Sequence similarity searches were undertaken using KEGG, NCBI and European Molecular Biology Laboratory (EMBL) platforms, with the associated Clustal tools (W, 2.1, and Omega, https://www.ebi.ac.uk/Tools/msa/clustalo/) for pair-wise and multiple sequence alignment. Domain analysis employed the NCBI conserved domain database (CDD) [78], the KEGG common motif (pfam) search tool in BLASTp and the UniProt platform [79]. Phylogenetic trees were constructed using the KEGG phylogenetic tree pipeline ETE3 [80,81] with default settings [82]. The layout of genes in sequenced genomes was determined using KEGG (gene map) and the Integrated Microbial Genomes and Microbiomes (IMG) platform of the Joint Genome Institute (JGI, Walnut Creek, CA, USA) (https://img.jgi.doe.gov/, most recently accessed on 10 February 2019). Guanine-cytosine (GC) content was calculated using the Endmemo DNA/RNA GC content calculator (www.endmemo.com/bio/gc.php, most recently accessed on 10 February 2019).

### 4.7. Protein Architecture Modelling

The UniProt platform [79] was used initially to identify topological properties of proteins, including the presence of signal peptides, coiled regions, transmembrane signatures and catalytic domains. Kyte and Doolittle [83] hydrophobicity plots based on FASTA sequences from UniProt were performed using the Bioinformatics Resource Portal (ExPASy) of the Swiss Institute of Bioinformatics (SBI, https://web.expasy.org/protscale/, most recently accessed on 10 February 2019) with window size of 9. The Phyre2 web portal for protein modelling, prediction and analysis [84] was employed to generate 3D protein models using intensity mode: protein sequences were resubmitted on multiple occasions to confirm architecture and models with >90% of residues modelled at >90% confidence submitted to the 3DLigandSite server to predict potential ligand binding [85]. Models were visualized using the UCSF Chimera program [86] with default settings.

## Supplementary Materials

Supplementary materials can be found at https://www.mdpi.com/1422-0067/20/7/1610/s1.

## Abbreviations

CDD	Conserved Domain Database (of NCBI)
CHAP	cysteine, histidine-dependent amidohydrolases/peptidases
GIT	gastrointestinal tracts
GC	Guanine + cytosine content of DNA
LAB	lactic acid bacteria
NSLAB	Non-starter LAB
PG	peptidoglycan
