# Supplementary material for: An Acid Up-Regulated Surface Protein of Lactobacillus paracasei Strain GCRL 46 is Phylogenetically Related to the Secreted Glucan- (GpbB) and Immunoglobulin-Binding (SibA) Protein of Pathogenic Streptococci
Source: Int J Mol Sci. 2019 Mar 31;20(7):1610. doi: 10.3390/ijms20071610 (PMC6479570; doi:10.3390/ijms20071610)
Supplement: Supplementary file 1 [file ijms-20-01610-s001.zip › ijms-453386/ijms-453386-supplementary materials.docx]

Pepper, S.J.; Britz, M.L. An Acid Up-regulated Surface Protein of *Lactobacillus paracasei* Strain GCRL 46 is Phylogenetically Related to the Secreted Glucan- (GpbB) and Immunoglobulin-binding (SibA) Protein of Pathogenic Streptococci

**Supplementary Materials**


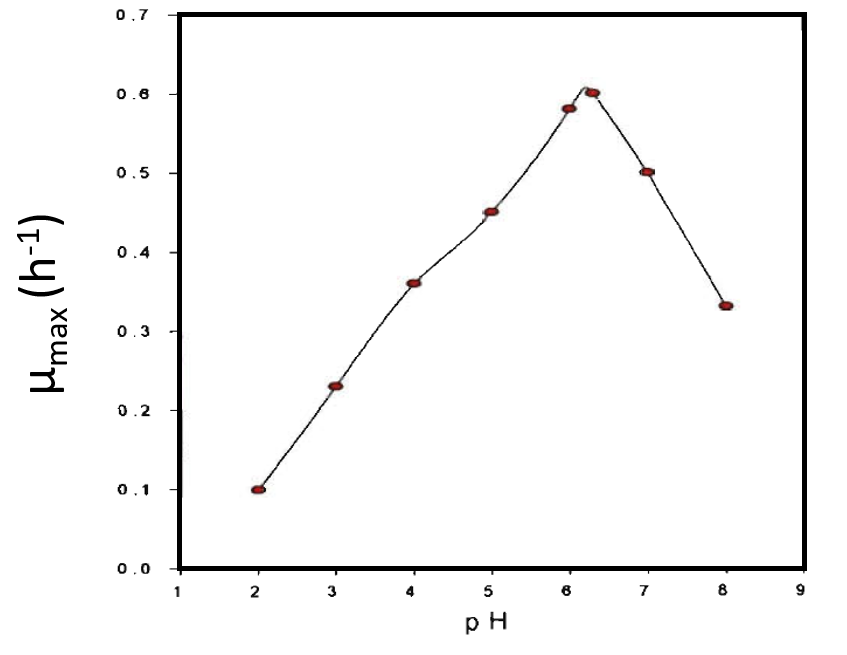


**Figure S1**. Maximum specific growth rate at different pH values. *L. casei* GCRL 46 was cultured in 1 L MRS broth in fermenters at 37 °C with set pH at 2 to 8, with nitrogen gas sparged at 0.1 L/min to maintain anaerobic conditions and set pH maintained by automatic addition of HCl or NaOH.


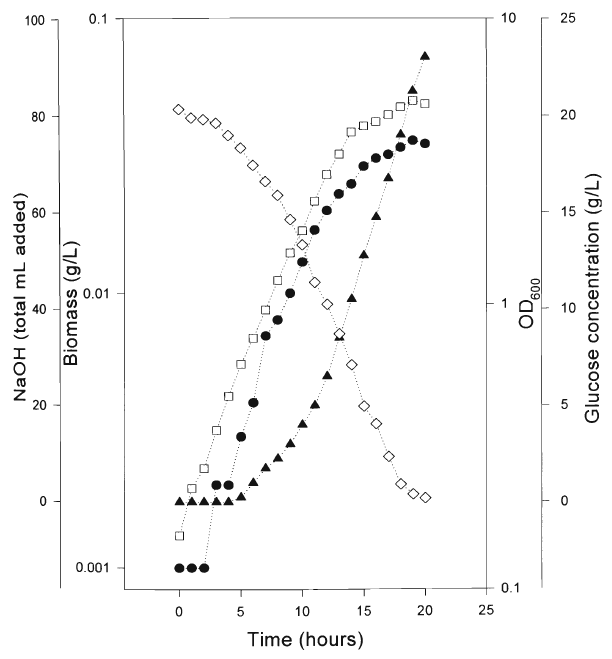


**Figure S2**. Growth kinetics of *L. casei* GCRL 46 under optimum pH and temperature conditions in fermenters. Growth was monitored by measuring OD_600_ (□), biomass (●), glucose utilization (◊) and volume of NaOH added to maintain the set pH at 6.3 (▲). The starter culture was an overnight culture in MRS broth, with cells concentrated 10-fold in fresh MRS prior to inoculation into fermenters to give a starting OD_600_ of 0.13-0.23.


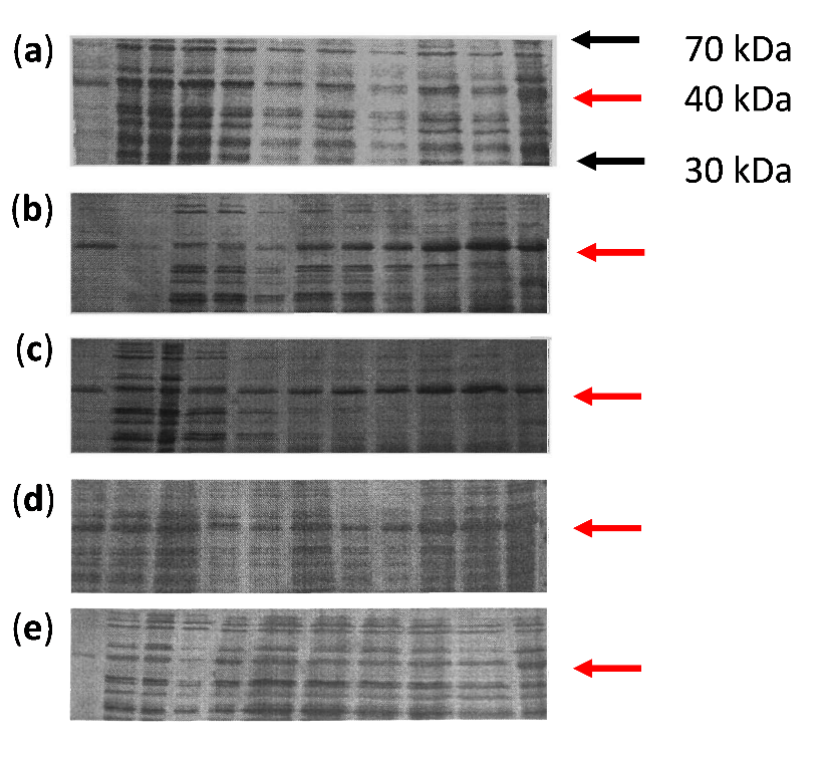


**Figure S3**. Images of 1D-SDS-PAGE gels for *L. casei* GCRL 46 cultured under different conditions of pH, for whole cell extracts sampled over time-courses of culture in fermenters. The images show focus areas of gels in the size range of 30-70 kDa for samples, as indicated in (a), and the red arrow shows where a protein of approximately 40 kDa would run. The starter culture was an overnight MRS flask culture without pH control, with cells concentrated prior to inoculation into MRS broth in fermenters to give a starting OD_600_ of 0.13-0.23. Samples were taken immediately after inoculation then for 9 h (lanes 1-10); the last lane is for a sample taken at 24 h. The culture conditions were:

(a) pH 6.3 until 9 h, then no pH control;

(b) culture at pH 4.0 throughout the culture period;

(c) initial pH 6.3, shift to pH 4.0 after 2 h culture, pH maintained at pH 4.0 until 24 h;

(d) initial pH 6.3, pH not controlled after inoculation;

(e) initial pH 6.3, shift to pH 4.0 after 2h culture, then returned to pH 6.3 for remainder of culture.

In (c), it took approximately 10 min to reach pH 4.0 and in (e) it took 20 min to cycle to pH 4.0 then back to pH 6.3. The data indicate that upregulation of proteins in the size range 40-45 kDa is linked to low pH growth conditions.


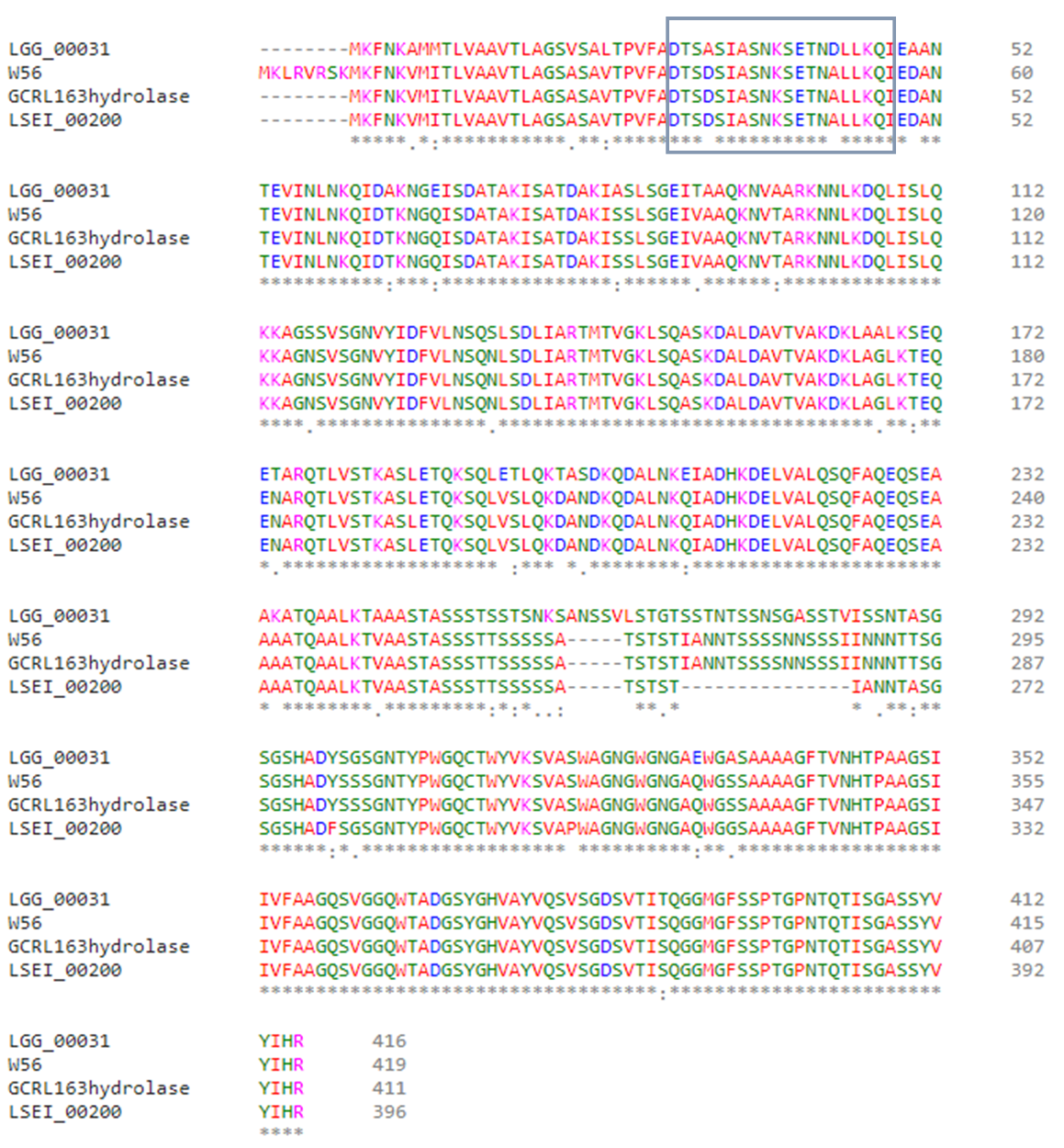


**Figure S4**: Sequence alignment for selected CHAP-domain containing *L. casei* group proteins. The boxed region depicts the sequence obtained by N-terminal sequencing of a protein in the MW range of 40-45 kDa that was seen on SDS-PAGE gels as up-regulated during growth at pH 4 in strain GCRL 46. Abbreviations:

LGG_00031 = *L. rhamnosus* GG, p40 surface antigen;

W56 = *L. casei* gene BN194_00240, possible TrsG protein;

GCRL163hydrolase = the protein identified by searching the genome of *L. casei* strain GCRL 163 using the N-terminal sequence;

LSEI_00200 = *L. paracasei* ATCC 334, surface antigen.

All proteins in this clade (see Figure 3b) contained the CwlO region often found in peptidoglycan DL-endopeptidases (orthology identifier K2147) and a CHAP motif.


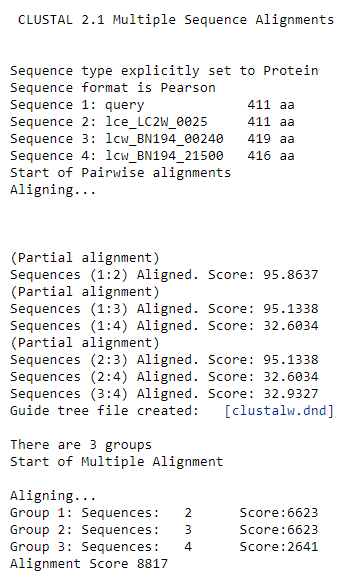

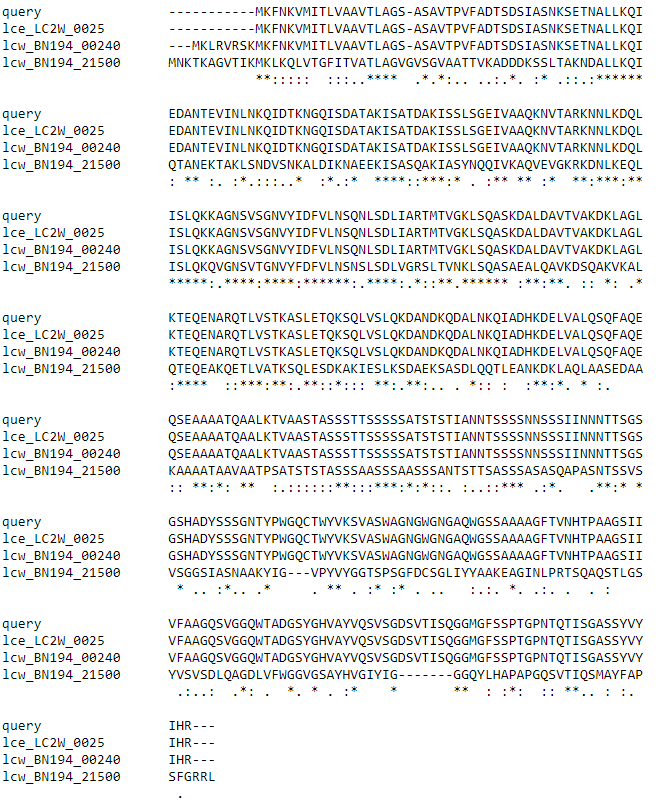


**Figure S5.** Sequence alignment for CHAP- and NLPC_P60-domain proteins in *L. casei*/*paracasei* strains. The query sequence was the TrsG protein from GCRL 163; LC2W_0025 and BN194_00240 (strain W56) are CHAP proteins, BN194_21500 contains the NLPC_P60 domain.

**Figure S6.** Layout of genes in plasmids and *Lactobacillus* species genomes around *trsG* genes (peptidoglycan DL-endopeptidase proteins with CHAP and NLCP_60 domains). Proteins specified by the gene locus numbers in the diagrams can be found in UniProt and KEGG databases. Endopeptidase gene locus numbers are boxed in red.

1. Layout of genes around the *trsG* gene AF91_15555 which specifies a protein of 825 AA in *L. paracasei* N1115.


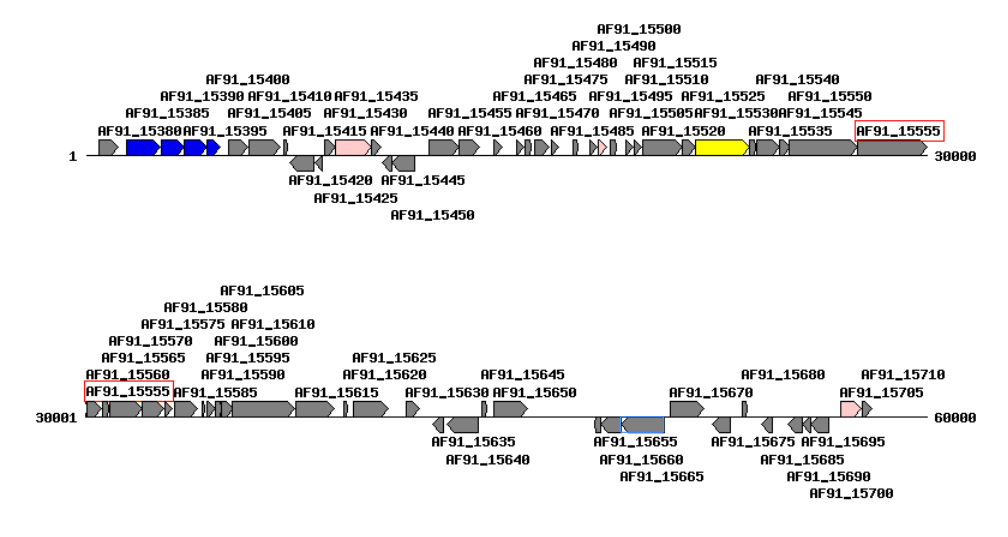


This is a plasmid, ca. 60 Kb, and has the signatures typical of plasmids found in *Lactobacillus casei* group plasmids: transposases, plasmid replication initiation protein, copy number control, integrase, permease and DNA-directed DNA polymerase, as well as many hypothetical proteins.

| Function | Gene ID number (AF91_ | Other information |
| --- | --- | --- |
| Transposases | 15420, 15425, 15580, 15585, 15620, 15625, 15635, 15670, 15675 (IS30) |  |
| DNA-directed DNA polymerase | 15435 |  |
| Copy number control | 15450 |  |
| Plasmid replication initiation protein | 15455 |  |
| Type IV secretion system | 15530 | VirD4, TraK, K03205 |
| Permease | 15535 |  |
| Integrase | 15660 |  |

1. Layout around plasmid-specified *trsG* gene of W56 (protein 825 AA, BN1940110) (flipped 180° to align orientation with the above layout). While the layout is not identical, the genes immediately around this region (upstream and downstream of the target) contain genes for phage replication proteins, integrase, phage terminator-associated genes and transposases associated with specific insertion elements (IS), similar to those in (a).


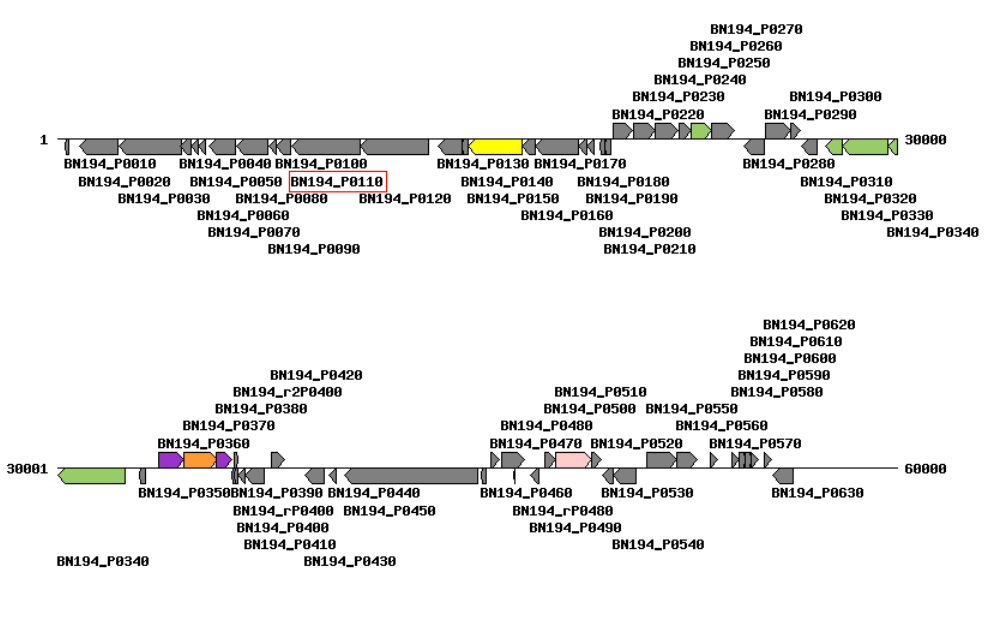


1. Layout for *L. paracasei* subsp. *paracasei* JCM8130, LBPC_2403 (325 AA, MW 34.8 kDa, sibA/CHAP domain and orthology identifier K21471. The protein has transmembrane signatures but not the CwlO motif


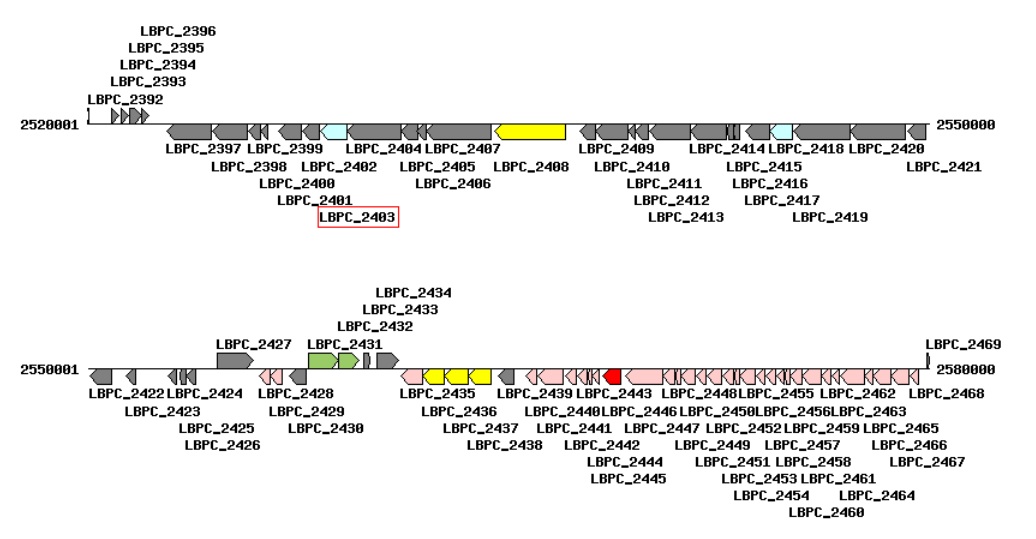


This region also has VirD4, TrwB/TraG_C and transposases the around this gene. The group of pink genes specify ribosomal proteins and other elements involved in protein synthesis (elongation factors etc).

1. *L. casei* W56 layout around the gene specifying the CHAP/CwlO1 protein. Phage-associated genes are located in the region from BN194_30180. A transposase is located at BN194_00550.


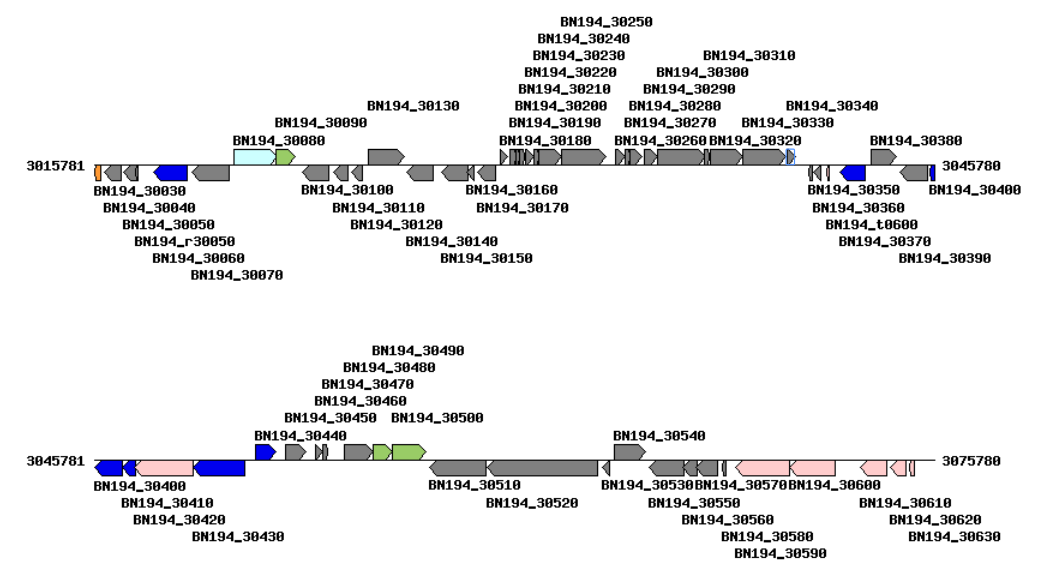


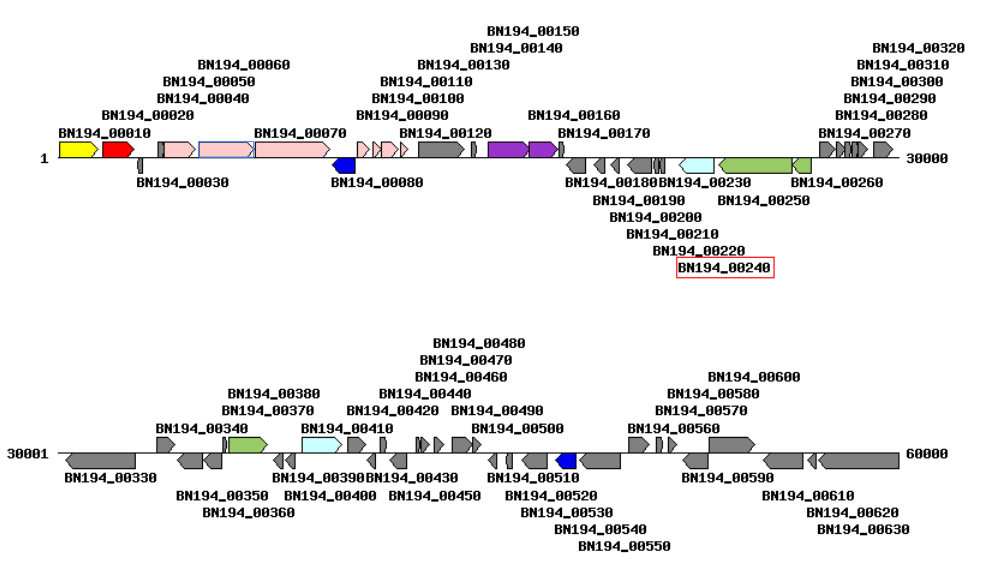


1. Layout around SibA/CHAP gene of *L. casei* GCRL 163 (from scaffolds available in the IMG database).


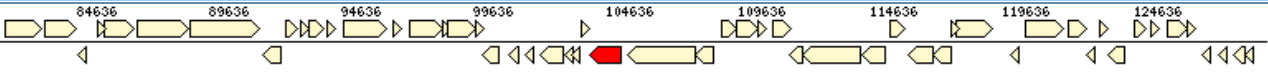


Note that the region ‘upstream’ of the origin of replication in W56 and GCRL 163 contains remnants of a prophage, indicated by genes specifying capsid proteins, replication and phage packaging machinery.

1. Layout around the gene specifying an NLPC_P60-CwlO1 protein of *L. casei* W56


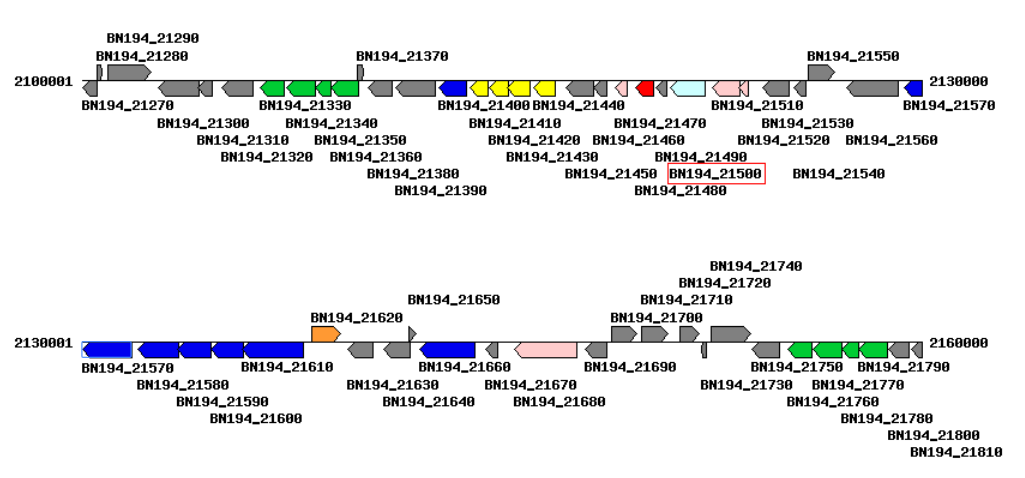


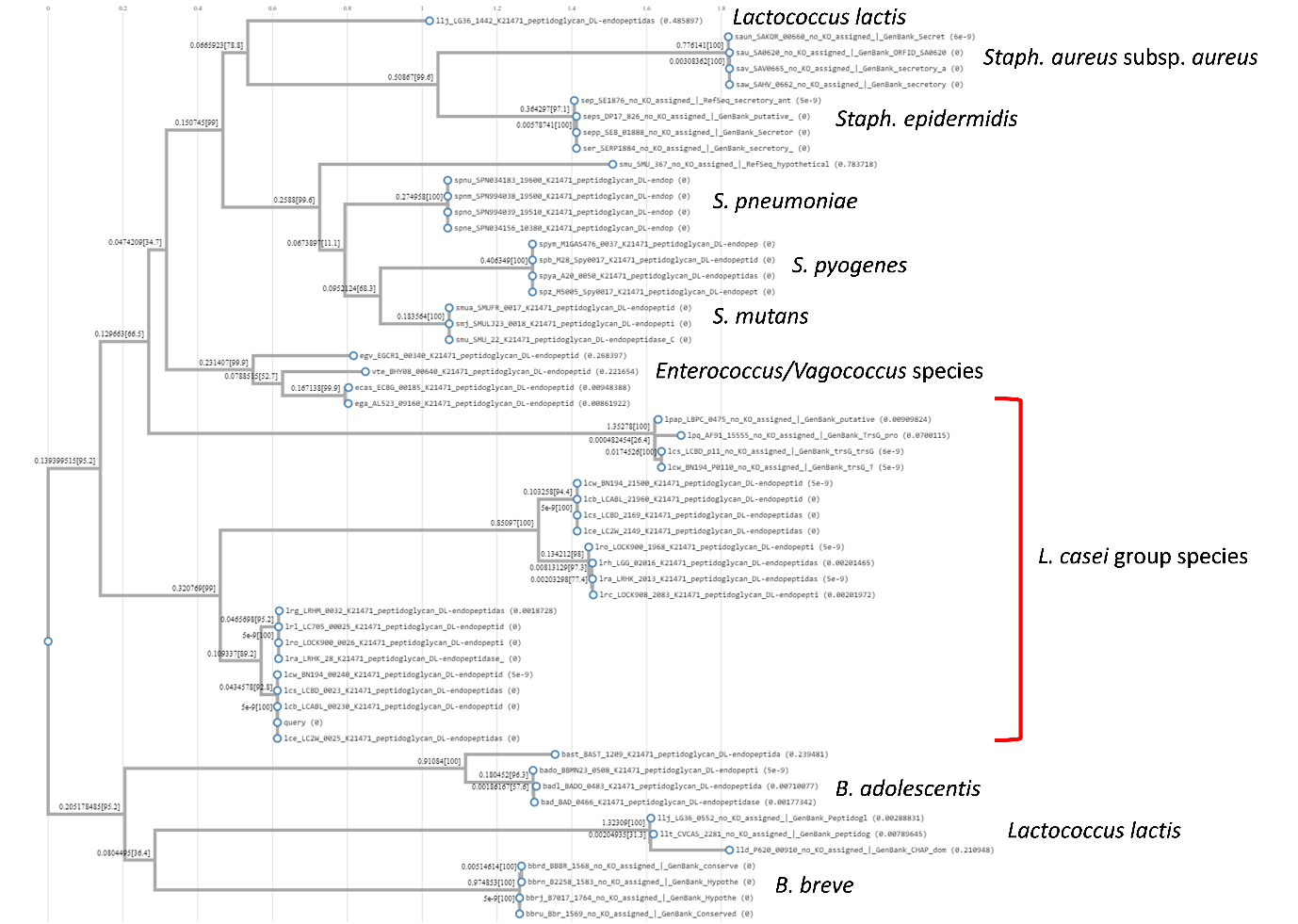


**Figure S7**. Mid-pointed rooted phylogram for selected species using the CHAP-ClwO1 acid-upregulated protein as query, for top 500 sequence similarity hits. Three to 5 examples of each species were selected from each genus to demonstrate broader phylogenetic relationships with the *L. casei* group protein (equivalent to BN194_00240). The *Staph. aureus* proteins have LysM-CHAP domains, the *Staph. epidermidis* have CHAP domains.


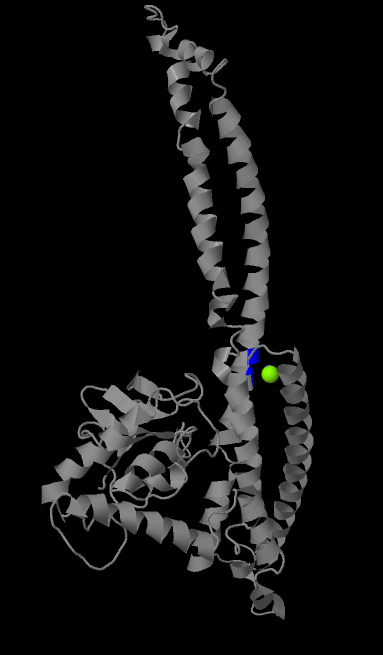

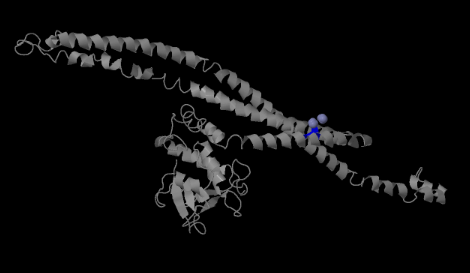

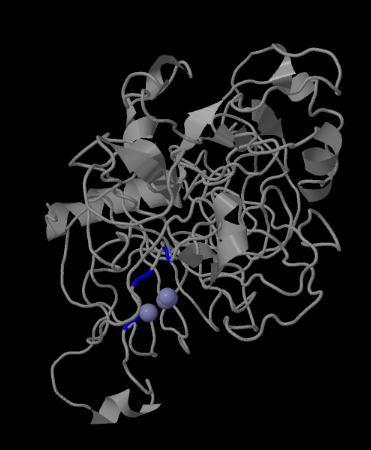

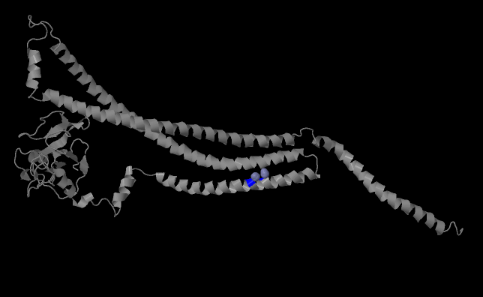


**Figure S8**. Examples of predicted ligand binding for selected modelled hydrolases. Left to right: *L. rhamnosus* NLPC_P60-CwlO1 protein, gene locus LRHMDP_K8QHD1, Zn and Mg site; *L. casei* NPLC_P60-CwlO1, gene locus BN194_21500, two Zn sites; *L. acidipiscus* LysM-CHAP protein, gene locus LAC1533_0489, two Zn sites; *L. casei* CHAP-CwlO1 protein, gene locus BN194_00240, two Zn sites.
